# Supplementary material for: The interactome of intact mitochondria by cross-linking mass spectrometry provides evidence for coexisting respiratory supercomplexes
Source: Mol Cell Proteomics. 2017 Dec 8;17(2):216–32. doi: 10.1074/mcp.RA117.000470 (PMC5795388; doi:10.1074/mcp.RA117.000470)
Supplement: Supplemental Data [file supp_RA117.000470_133922_0_supp_23978_fzffwf.zip › spectra_annotation/mito_spectra_annotation/147-1-8-1-16-1.pdf]

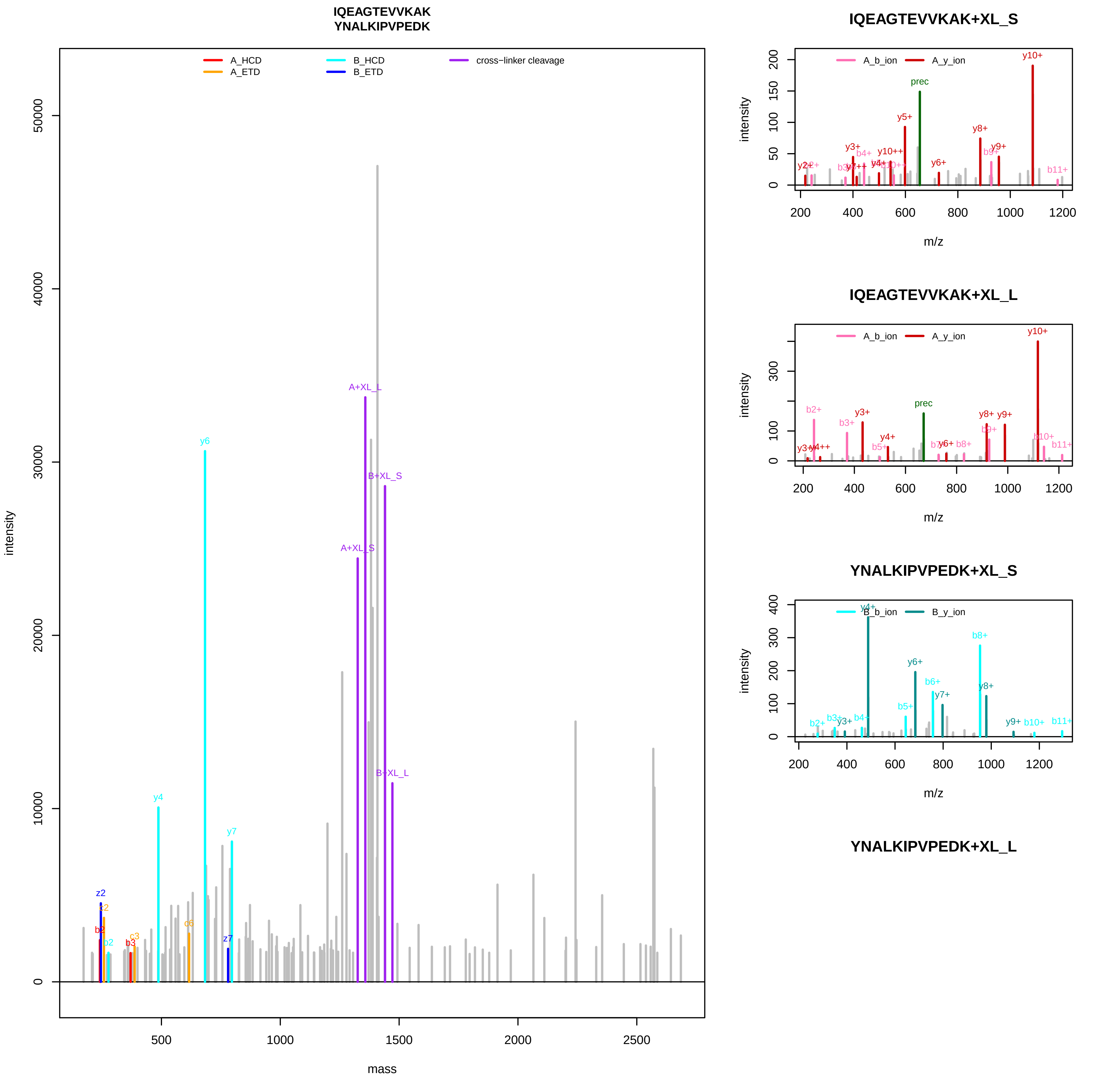

IQEAGTEVVKAK
YNALKIPVPEDK
A_HCD
A_ETD
B_HCD
B_ETD
cross−linker cleavage
intensity
mass
A+XL_L
B+XL_S
A+XL_S
B+XL_L
y6
y4
y7
z2
c2
b2
b3
c3
c6
z7
IQEAGTEVVKAK+XL_S
A_b_ion
A_y_ion
prec
y10+
y5+
y8+
y9+
y3+
y6+
b11+
m/z
IQEAGTEVVKAK+XL_L
b2+
b3+
y4+
b5+
y8+
b10+
YNALKIPVPEDK+XL_S
B_b_ion
B_y_ion
y4+
b8+
y6+
b6+
y7+
b5+
y3+
b4+
y9+
b10+
b11+
YNALKIPVPEDK+XL_L
